# Epistemic Health, Epistemic Immunity and Epistemic Inoculation

**DOI:** 10.1007/s11098-023-01993-9

**Published:** 2023-06-02

**Authors:** Adam Piovarchy, Scott Siskind

**Affiliations:** 1 The Institute for Ethics and Society, The University of Notre Dame, Australia, Sydney, Australia; 2 San Francisco, USA

**Keywords:** Epistemic health, Social epistemology, Trust, Inoculation

## Abstract

This paper introduces three new concepts: epistemic health, epistemic immunity, and epistemic inoculation. Epistemic health is a measure of how well an entity (e.g. person, community, nation) is functioning with regard to various epistemic goods or ideals. It is constituted by many different factors (e.g. possessing true beliefs, being disposed to make reliable inferences), is improved or degraded by many different things (e.g. research funding, social trust), and many different kinds of inquiry are relevant to its study. Epistemic immunity is the robustness with which an entity is resistant to performing certain kinds of epistemic activity, such as questioning certain ideas, believing certain sources, or making certain inferences. Epistemic inoculation occurs when social, political or cultural processes cause an entity to become immune to engaging in certain epistemic activities. After outlining each of these concepts, we close by considering some of the risks associated with attempts to improve others’ epistemic health.

## Introduction

During the COVID-19 pandemic, a number of hurdles made getting people vaccinated difficult. After overcoming the challenges of creating, producing, and distributing effective vaccines, a recurring problem was that many citizens didn’t trust official medical advice. A variety of easily debunked reasons were cited for their hesitancy: the vaccine was going to control people’s minds, kill people, or lead to government tracking (Islam et al., 2021). Some also believed that the pandemic either didn’t exist or was a conspiracy in service of other nefarious ends (Goodman and Carmichael, 2020). But many people’s lack of trust seemed to have a less clearly defined cause, manifesting more as a feeling or suspicion rather than clearly identifiable reasons (Tomljenovic et al., 2020).

For epistemologists and folk alike, there’s a variety of ways we can understand and evaluate various states of affairs, such as when large numbers of people no longer trust experts. One can obviously ask whether such states of affairs are good or bad. One can also analyse whether such decisions are rational, given individual preferences and expected costs (Böhm et al., 2016). Alternatively, one can analyse whether citizens’ beliefs are justified, given their available evidence. Another lens from which to evaluate such states of affairs is to consider the contribution of virtuous and vicious intellectual traits (e.g. Cassam 2018). And social epistemologists now regularly examine whether certain arrangements—such as who is deemed sincere or trustworthy—are just or unjust (Fricker, 2007).

Recently, however, a number of philosophers have begun using terms to describe and evaluate epistemic states of affairs that don’t quite fit into any of the above options. Joshi (2022), for instance, describes social pressure to adopt certain political stances as “bad for our collective epistemic health” (p. 397) because it prevents valuable, distributed evidence from being contributed to the commons where it can be collated. When describing people who are stuck in echo chambers, dismissing any new evidence that they should change their beliefs, Begby (2021) writes their “predicament is pathological… these people are obviously not leading healthy and prosperous cognitive lives” (p. 525). Aird (2022) has claimed that various instances of misinformation are an “obvious instance of a government sacrificing its peoples’ epistemic health for political gain” (p. 9). Other examples of similar terms being used by social epistemologists abound (Brown, 2019, p. 144; Habgood-Coote 2019; McKenna, 2020, p. 102; Solomon & Richardson 2005, p. 211; Sullivan et al., 2020, p. 740). Less recently, Williamson (2005), in an engaging introduction to the pitfalls of scepticism, has described scepticism as a “disease” of our “cognitive immunity system” (p. 681). According to Merritt (2014) even Kant “thinks that the ‘correct use’ of the power of judgment… can be conceived as nothing other than ‘healthy understanding’” (p. 138). As we’ll see, there is also an entire field of psychological research currently trying to understand the ways that people form and update their beliefs in health-related terms.

None of these authors give much treatment of these terms, typically mentioning them only once in an off-handed fashion.^1^ We think these terms are not mere rhetorical flourish, but are getting at ideas and concepts that are actually quite useful. While it already easy enough to understand each author’s meaning, a more explicit analysis of these concepts (and some we shall propose) can aid social epistemologists and citizens in understanding, analysing, and evaluating different entities and states of affairs, given our interest in multiple epistemic goods and the myriad ways in which they can be promoted or compromised.

What makes a concept useful is subject to a range of considerations: the ease with which audiences can understand it, the extent to which it enables communication or simply leads to more confusion, whether it does any ‘joint carving’, whether it serves our political goals, etc. We don’t intend to adjudicate this matter here, or put much emphasis on explicit desiderata. Instead, we shall simply outline the concepts, how we think they should be understood, some of their uses, and expect that many readers will find them intuitive and useful, evidenced partly by the fact that they are *already* being used and understood.

Here are our proposals: *Epistemic health* is a measure of how well entities (e.g. persons, communities, institutions) are functioning with regard to a range of epistemic goods or ideals. *Epistemic immunity* is the robustness with which one is resistant to engaging in certain kinds of epistemic activity, such as questioning certain ideas, believing certain sources, or making certain inferences. *Epistemic inoculation* occurs when social factors cause an entity to become immune to engaging in certain epistemic activities. These concepts are developed by building on our existing concepts of health, immunity and inoculation, and the role they currently play allowing us to analyse, evaluate and improve our physical well-being. After outlining these concepts and their usefulness, we close by considering some of the risks associated with attempting to improve our epistemic health.

## Health

To understand epistemic health, it will help to first make some observations regarding the concept health, its different conceptions, extensions, roles, and our use of its associated term. Health, understood as a property, can be satisfied by a range of objects. We speak of healthy individuals, healthy communities, healthy nations, and healthy ecosystems. Sometimes we understand health as applying within a certain domain, such as physical health, mental health, or emotional health. Multiple factors can be relevant within each of these domains, even within a single individual. When speaking of someone’s physical health, this can include their immune system, blood pressure, and organs. Much of the above also applies to talk of an absence of health. We talk of unhealthy individuals, communities, and ecosystems.

A variety of factors also cause health to improve or decline. Too much sodium causes high blood pressure, and load-bearing exercise causes one’s muscles to grow stronger. But many factors also *constitute* good health; lungs which are not properly exchanging oxygen doesn’t just cause one to be unhealthy, it’s part and parcel of what makes it the case that one is unhealthy. The qualities that make something healthy or unhealthy can themselves possess a range of different properties. We speak of *strong* hearts, *resilient* immune systems, *elastic* arteries, *high* oxygenation.

Health can be analysed by traditional conceptual analysis. We could say that one has health if and only if one has normal species functioning.^2^ Health can also be understood in negative terms: Boorse (1977) proposes we understand it as the absence of pathology. However, it is more common to think of health as a cluster-concept without necessary or sufficient conditions, and there are a variety of ways we can understand the things that fall within its scope. We can examine what factors are more paradigmatic, central, or weighted more heavily with respect to our health assessments, in contrast to those that are more contingent or peripheral. Health ascriptions can also be context-sensitive or relative. We often say that someone is healthy given what their prognosis was five years ago, or healthy for an 80-year-old. Social factors can lead to socially-constructed conceptions of health (Olafsdottir, 2013). Regardless of how much you personally value fitness, you might come to think that fitness is healthy because this is what society has deemed to be healthy.

Our ideas of health and uses of the associated concept are often normative. Being healthy is often considered desirable or valuable, motivating us to promote particular ends, and health plays a valuable role helping us co-ordinate to produce those ends. We encourage others to be concerned with their health, we develop medical systems to restore health, and we have public health campaigns aimed at preventing ill health. Conversely, what states we think are valuable can, in turn, influence what we think of as healthy. Someone who values living to as old an age as possible might see a diet that foregoes many foods as healthy, while someone who values variety in their diet might see such a restrictive attitude as pathological. Finally, our applications of the concept are often implicitly prescriptive. For example, when we say smoking is unhealthy, this is not purely descriptive, like when we say that cigarettes contain plant matter. We mean that smoking is *bad*, and one has reasons to abstain from it.^3^

In some contexts, people take themselves to be using ‘health’ in value-free ways. When you speak of a fungus being healthy, you probably don’t impute any kind of value judgement about this since you don’t care about most fungi. Scientists often speak of the poor health of an eco-system as being part of the cause of its collapse, or the good health of an organism being part of the reason that its genes were passed on, and take themselves to be making descriptive claims.^4^

This variety of ways of understanding health leads to very different kinds of academic inquiry. Nevertheless, differing research programs can have sufficient overlap to be usefully considered as operating within the same domain in a way. Now that we’ve got an idea of what health is and how this concept gets used, we can understand what makes it useful.

First, the concept health enables us to *evaluate* many things in ways that are helpful given our interests or the things we care about. Knowing that smoking is unhealthy and that exercise is healthy greatly helps us decide how to spend our Sunday afternoons, given our desire to live a long life, even if we don’t understand how exercise or smoking affects our bodies as well as, say, a scientist does. It also lets us identify the moral qualities of many actions in virtue of their effect on others’ health. Knowing that dumping toxins in the water supply will negatively affect people’s health gives us strong moral reasons to avoid doing such a thing.

Second, it enables a lot of *co-ordination*. Given many of us have strong interests in remaining in the kinds of states commonly picked out as healthy (e.g. alive, nourished, not sleep-deprived), and given we often need to co-ordinate to produce environments in which those states obtain (having food systems that don’t poison us; making sure workplaces don’t have 40-hour shifts), the concept helps us collectively work in ways that are valuable. Being able to identify e.g. that smoking is unhealthy makes salient the risks to any given individual smoking and the lost utility that comes from allowing smoking as a society, draws upon shared social norms about what things should not be done to motivate others to avoid smoking, and enables people (who may not know a lot about carcinogens but who have shared concerns) to identify common relevant features of the world and work together.

Third, it enables us to *understand* something about the world, typically something to do with the state or functioning of various systems, both intra- and extra-personal. The concept health can also allow us to understand why some things are bad beyond the bare fact that e.g. we desire to avoid them. For example, it enables us to see the fact that arsenic is poisonous as something which coheres with a backdrop of other facts about our biology, evolutionary history, and why our bodies function as they do, rather than a single, odd, detached fact about the world. It enables us to predict that poison will also have certain effects on other animals, and to see that that many of the things which are picked out as poisonous have some kinds of similarities that are worth tracking.

Understanding the world, evaluating it, and co-ordinating with others are pretty good uses for a concept to have. There is a lot more we could say about the concept health and its different conceptions, extensions, cognates, associated concepts, and usefulness, but we’ve said enough to understand the roles it plays for us. We believe there is another domain in which a new concept could play similar roles. It is with this goal in mind that we would like to introduce the concept of epistemic health.

## Epistemic Health

All of what was said in the previous section is just as true of epistemic health, which we propose be understood as a measure of how well an entity is functioning with regard to a range of epistemic goods or ideals. Epistemic health can be possessed by a range of objects. We can talk meaningfully of epistemically healthy individuals, families, communities, networks, nations, and systems. Epistemic health can be assessed within a range of domains, such as the set of one’s beliefs, the inferences one is disposed to make, or one’s cognitive capacities. Each of these is in turn made up by a range of things. Agents might have healthy priors, healthy scepticism, or a healthy capacity for theoretical reason.

We can also talk meaningfully about objects that have poor epistemic health, and what makes it the case that an object has good or poor epistemic health can be determined by a range of factors. Perhaps a community has *corrupt* knowledge-producing institutions (e.g. academic or media outlets), *blocked* informational networks (government censoring a media outlet; no connections to alternative viewpoints), *unreliable* community agents, or *low* levels of social trust. Many factors can cause epistemic health to improve or decline. Well-resourced academic institutions can increase the knowledge that the community has; social pressure can prevent certain viewpoints from being disseminated or considered. Some factors arguably constitute good epistemic health: that a community has lots of true beliefs that are caused in the right sort of way doesn’t just cause them to have good epistemic health, it partially constitutes good epistemic health.

Epistemic health could, in principle, be understood through traditional conceptual analysis, though we are not aware of any attempts given we are proposing it here as a new concept.^5^ It can also be understood as a cluster-concept, and we can examine what features are more central or paradigmatic or weighted more heavily, in contrast to those which are more peripheral. Some beliefs seem more important to our epistemic well-being than others and thus we might have more interest in processes producing those beliefs rather than others; how reliably or accurately an agent or community forms beliefs about political matters seems more central to our epistemic health assessments than how reliably or accurately they form beliefs about grass, for instance.^6^ Ascriptions of epistemic health can be context-sensitive or relative. A community might have good epistemic health relative to some reference class, or given some obstacle that they’ve had to overcome, while still having poor epistemic health according to some other measure.

Ascriptions of epistemic health are often going to be value-laden in a variety of ways. Given people often think that e.g. having true beliefs is both instrumentally and non-instrumentally desirable or valuable, this will cause us to promote certain ends. We might want to fund certain kinds of research, or to create laws limiting censorship, if we think these are the means that will effectively produce the kinds of epistemic activities we care about. Second, what kinds of states we think are valuable can influence what we think of as epistemically healthy, independent of whether those states are in fact epistemically healthy or not. For example, given you think that endorsing Cartesian scepticism leads to missing out on all kinds of epistemic goods, you might think that someone who lives according to Cartesian scepticism is epistemically unhealthy, while they might think the epistemic risks you are taking in admitting beliefs you are not absolutely certain of is unhealthy. Third, our ascriptions of epistemic health are often implicitly normative. Saying that conspiracy theorists often form unreliable beliefs on the basis of a selective and inaccurate interpretation of evidence is typically much more negatively valanced than saying that conspiracy theorists often eat food, for instance.

In some contexts, talk of epistemic health could also be used in relatively value-free ways. We could speak of some networks having higher epistemic health than others while remaining neutral on whether this is a good or bad thing, and we can pick markers of epistemic health that don’t seem to depend in any way upon our particular values. We might make such claims because they are epistemically fruitful, helping us explain certain phenomena and predict future events.^7^

It should be clear that the concept epistemic health is useful in ways that are very similar to that of the concept of health. First, the concept helps us *evaluate* many things in ways that are helpful given our interests or the things we care about. Once one has the concept of epistemic health, one can now assess the epistemic functioning of individuals, communities, networks, countries, or institutions. One can consider the merits of various proposals. At least, it enables us to identify a kind of benefit or cost that can be weighed against other costs and benefits we care about. For example, a policy might improve epistemic health, but lead to lower happiness.

Second, the concept helps us co-ordinate. Given many of us care about the epistemic health of ourselves, our families, our communities, and our country, identifying that a certain policy improves or degrades their epistemic health can help us work out how to avoid or engage in that policy. One can initiate and direct new forms of inquiry that may have otherwise been too nebulous.

Finally, the concept helps us understand the world in certain ways that seem practically and theoretically useful. We can interpret a political scientist seeing what improves people’s voting patterns, and a philosopher teaching students about common errors in reasoning, to be operating within a shared domain despite there being many differences between the two. We can provide macro-level explanations of phenomena, and identify causal relations between things that may not otherwise have been noticeable. Perhaps a community’s low compliance with public health orders, damaging policies, and spread of misinformation can be seen as all being attributable to the community’s poor epistemic health.

Once again, there is a lot more we could say about this concept, and the various kinds of inquiry that can fall within its scope. But before we move on to considering our other proposed concepts, let us address two worries readers might have. The first is that our proposal may seem to have some superficial similarities to virtue epistemological approaches. Reliabilists have historically had an interest in functioning, for instance, and virtue epistemologists have argued that, in addition to individuals, institutions and collectives can also be virtuous or vicious. A full examination of this issue is outside the scope of this paper, but let us make a few remarks. One clear difference regards masks. It is well-known that many things can mask a virtue from manifesting, without thereby counting against its attribution to an agent. Someone might have perfectly virtuous epistemic traits, for instance, but find themselves in an adversarial epistemic environment that has been set up to specifically to deceive. However, many kinds of factors which mask virtue are instead likely to *undermine* your epistemic health, at least in some respects. Additionally, much of the interest in functioning historically comes from reliabilists interested in particular faculties and matters such as whether someone’s belief counts as knowledge or warranted, and these seems to be rather different interests with different standards than ours. Knowing that someone is healthy and functioning quite well, epistemically speaking, tells us little about e.g. whether they have knowledge or warrant, and knowing that e.g. their Gettierised belief is knowledge because it stems from properly functioning faculties tells us quite little about their epistemic health.^8^

We also think our framework has a higher chance of uptake within some communities, given most people already possess health-related concepts but lack background in character traits and dispositions (e.g. students in a critical thinking class, or an introduction to statistics class learning about the replication crisis). For the uninitiated, hearing that existing scientific practices are manifesting epistemic vice can sound antiquated or like an attempt to be poetic. Hearing that scientific practices are not as epistemically healthy as they should be seems, to our ears, likely to get the intended meaning across immediately in an engaging manner. As already noted, these terms are also already being used by a variety of people, many of whom are perfectly familiar with virtue epistemology but who decided to not use its terminology. It’s not clear to us that their purposes would be better aided by pushing them to instead use the language of epistemic virtue and vice.

The second worry readers might have here is that most of its appeal seems to come from its ability to, within some domain, talk about a plurality of items thought to be valuable and which depend on some functioning system to be maintained, and that aside from this our existing concept of health is doing little additional work. This can be shown by noting the ease with which we could apply the term ‘health’ to virtually any domain which has a certain set of goods, and which requires some kind of functioning to produce those goods. For example, we could also speak of ‘artistic health’, which could apply to individual artists and communities, and which is concerned with how well various items thought to be valuably (e.g. quantity of artworks, quality of artworks, understanding of art in the community, status of art and artists in the community). We could also speak of ‘sporting health’, ‘housing health’, ‘office health’, and so on. And at this point there may be little benefit talking about ‘epistemic health’ rather than ‘epistemic functioning’.

We are sensitive to this worry, but think it can be overcome. One quick rejoinder is to point out that the domain of epistemology’s subject matter is much more explicitly or substantively normative than say, office arrangements. We have epistemic norms (Henderson & Graham, 2019), we routinely engage in epistemic evaluations (Henderson & Greco, 2015), we hold others epistemically responsible (Peels, 2016) and experience epistemic blame (Piovarchy, 2021). In short, there seems to be an important source of epistemic normativity that is not merely hypothetical in nature (i.e. ‘*if* you want to reason well…’; Grimm 2009). ‘Health’, being already often understood as normative or a practice- or mind-independent property, helps capture this quality more than ‘functioning’ does, which applies too easily to domains that arguably have no value at all (e.g. one can function very well as an assassin without this being good at all). We also seem to have much more reason to be concerned with epistemic standards, given they help ensure that our division of epistemic labour produces important collective epistemic goods (Brennan, 2010). But a more convincing way to show there is utility from making an analogy with our existing health concept is to show this characterization opens up avenues for thinking about our epistemic world in ways that have similarities to other existing health-related concepts. This is what the remainder of this paper will do. In the next two sections, we consider some properties that are particularly relevant to our assessments of epistemic health, and which will also be useful for thinking about the epistemic health of particular entities: epistemic immunity and epistemic inoculation.

## Epistemic Immunity

What is needed to maintain good epistemic health? Many things will contribute. One will need access to certain resources, such as information. One will also need certain kinds of training, such as how to use said resources. But another relevant factor is that one remains free from damaging ideas, habits, or inferences. This is where the concept of epistemic immunity comes in. In medicine and biology, immunity refers to an organism’s ability to be “exempt or hav[e] resistance [which results] from responses that protect hosts from pathogens or other threats” (Mills et al., 2015, p. 444). Even if a patient doesn’t currently have influenza, if they have an ineffective immune system we’re going to be very worried about influenza precisely because they may be unable to fend off future attacks from it. Likewise, in thinking about one’s epistemic health, we don’t just want to know what one currently believe, it also matters what one would believe in certain situations. And some situations particularly relevant to our assessments are ones that involve certain *threats* or *pathogens*.

Epistemic immunity can be understood as one’s resistance to engaging in certain forms of epistemic activity, typically unhealthy ones (but not necessarily, as we’ll argue below).^9^ Examples of epistemic activity include forming certain kinds of beliefs, trusting certain sources, or making certain kinds of inferences. It is important to note that the strength of one’s epistemic immunity is a purely functional notion, measured in how robust one’s resistance to engaging in certain activity is across a range of relevant counterfactuals or relevant possible worlds. Being too inattentive, or arrogant, or prejudiced against a speaker to follow their sophisticated argument for an unhealthy conclusion can actually contribute to one’s immunity against accepting that argument. Such dispositions might still be bad for one’s overall immunity and overall epistemic health though, given the other threats they leave one exposed to.

One factor to keep in mind when considering epistemic health is the need for a broad understanding of what factors favour certain epistemic activities. We need to be mindful of factors such as higher-order evidence, which go beyond the content of someone’s argument. For example, you might not understand the reasons your doctor gives you to lower your cholesterol, but take the fact that it is your doctor (a relevant expert) telling you to do so to be a reason to do so. Many factors contribute to whether arguments are effective, or whether evidence is taken to count as evidence, beyond the evidence itself or the content of said argument.

This particularly matters for assessing whether particular dispositions are immunity-improving or immunity-compromising. A large body of research demonstrates that people’s reasoning (and with it, what they end up believing) are influenced by seemingly irrelevant factors like how easily they can think of examples, how stereotypical a description is independent of base rate information, or the way in which options are framed (Kahneman, 2011). Though such tendencies are commonly taken to be ‘biases’ in a pejorative sense, we shouldn’t be quick to assume that we would therefore be epistemically healthier or more immune to bad ideas without such tendencies. Numerous scholars have argued that many biases, heuristics, automatic processing, and ‘System 1’ reasoning can be *ecologically* rational, as they result from mechanisms which typically deliver correct judgments given the set of environments that one finds themselves in (e.g. Neth & Gigerenzer 2015). For example, while being disposed to vote for whichever candidate is listed first on a voting form seems like a paradigmatic instance of irrationality given listed order does not correlate with quality (Doris, 2018; Meredith & Salant, 2013; Levy, 2019) argues that the order in which options are presented is often interpreted as an implicit recommendation. And mechanisms which favour first-presented options (or cause us to believe that the first presented option is best) can, on the whole, be rational, if one is in an environment where people with good will do often present their implicit recommendation first.

Of course, in many cases our dispositions will deliver inaccurate results, and we often have strong moral reasons to improve our accuracy (Fricker, 2007). But we mention this to emphasise that when considering which dispositions or activities are worth cultivating or removing, we need to consider how they operate across many environments, not merely those in which they lead to one particular judgment we’ve identified as unjustified or unhealthy.

Epistemic immunity can also be a property of collectives such as communities or nations. Two different communities might have very similar beliefs, resources and reasoning capacities, and yet one might be much more *robustly* disposed to ward off threats due to other factors. To assess the epistemic health of communities, then, it won’t be enough to just look at how things are functioning currently. One will also want to check that the community has the kind of resources, institutions and structures necessary to maintain that functioning should circumstances change.

A particularly interesting example of why it is useful to think about the epistemic immunity of communities, and how the epistemic health of individuals and their communities can come apart, comes from Levy and Alfano (2020). Levy and Alfano detail how cultural knowledge can be created and sustained by individual habits that are epistemically vicious. Individual agents may behave in ways that are dogmatic, incurious, or arrogant, and yet which, over time, deliver genuine knowledge. This is often practically important knowledge which is adaptive for the community (e.g. enabling them to detoxify poisonous food for consumption), in which the relevant causal mechanisms are opaque (Henrich, 2015). While much of these beliefs are action-guiding—they are beliefs about which actions should or should not be done—much of this cultural knowledge is also belief-guiding: they are beliefs about what one should believe, or beliefs about what kinds of inferences or questions are legitimate or illegitimate. Norms against questioning authority and tradition, for instance, could plausibly serve to maintain systems that in fact improve the community’s epistemic health, but this benefit and the mechanism by which it is produced may not be well-understood by any one individual.

Such examples are important for thinking about epistemic health because they demonstrate how the epistemic health of a community can be better or worse than what one might infer by simply aggregating the level of epistemic health of each individual. If we focus on any one individual’s resources, skills, and activities, we will likely conclude that they are behaving in epistemically vicious ways, and perhaps that they have poor epistemic health. But by looking at how their epistemic activities function within the broader network, we can notice that certain kinds of activities are in fact conducive to the community’s epistemic health.

There’s a potential difference between how we think of biological immunity and epistemic immunity. We’ve said that epistemic immunity can typically be understood as resistance to engaging in certain unhealthy epistemic activities, but biological immunity is nearly always thought of as positive. People sometimes speak of an over-active immune system, but not of someone having ‘too much immunity’. Should the same convention be applied to talk of epistemic immunity? In principle, one could choose to describe most instances of robust resistance to healthy epistemic activities as ‘epistemic illness’, and reserve talk of ‘immunity’ for resistance that is deemed positive.

We think there are a few reasons to favour understanding epistemic immunity in neutral terms. In keeping with current usage, it is not uncommon to describe an interlocutor one is frustrated with as ‘immune to reason’, but this would be inapt if we took immunity to always be positive. Additionally, in many cases, the dispositions which lead to or constitute healthy resistance in one context will be the same dispositions creating unhealthy resistance in other contexts. It seems useful for researchers interested in such dispositions or mechanisms to think of them as single entities, rather than two distinct sets whose manifestation depends on context. Thinking of epistemic immunity in neutral terms is also likely to make it easier to talk about resistance in contexts where what counts as epistemically healthy is disputed. When seeking to describe a particular agent’s resistance to believing a certain idea, people risk getting into a verbal dispute over whether the agent is epistemically immune or epistemically ill. In contrast, thinking of immunity in neutral terms makes it easier for everyone to agree that the agent is immune to certain things, and leave it as an open independent question whether this is epistemically healthy or not. We thus propose that, all else being equal, or absent any other indicators, when a speaker picks out an agent or entity as being ‘epistemic immune’ to certain ideas, we consider them to be taking a neutral stance on whether this immunity is healthy or unhealthy.^10^

In the next section, we want to introduce one particularly interesting way in which epistemic immunity is produced, which has not yet been considered within the philosophical literature: epistemic inoculation.

## Epistemic Inoculation

In medicine, immunisation refers to a process by which an organism becomes immune to certain pathogens. The most well-known method of producing immunisation is inoculation: introducing pathogens to our body that our immune system is able to overcome, which then gives our immune system the ability to defend against stronger pathogens in the future.^11^ For example, the virus that causes measles can be deadly. But by presenting the immune system with a weakened version of this virus, the immune system recognises it as a threat, produces antibodies, defeats it, and from then on has ‘learned’ how to ‘recognise’ what the measles virus ‘looks like’, and how to defeat later, stronger versions before they have time to replicate and do damage. Importantly, for inoculation to be effective, the pathogen must be strong enough to be registered as a potential threat in order to produce antibodies, but weak enough to not overwhelm the body’s defences (Pollard & Bijker, 2021). Also worth noting is that the earlier, weaker pathogen need not be the same as the later, strong pathogen, to produce inoculation. For example, 20th century inoculation against smallpox was actually produced by administering a vaccine made from the vaccinia virus.^12^ All that matters is that the immune system ‘thinks’ the later pathogen is sufficiently similar to the earlier pathogen.

Epistemic inoculation occurs in a similar way. Here’s a first pass at the phenomenon we are interested in: by exposing an agent to weak reasons favouring some epistemic activity, which the agent is able to overcome or resist, the agent is then able to later defend against much stronger reasons favouring that activity. It is important here that when talking about the ‘strength’ of a reason, we do not mean its rational strength. Nor when we say an agent ‘overcomes’ or ‘defend against’ a reason do we mean that the agent is able to find reasons that are in fact good reasons to discount or ignore the first reason. We mean this in purely functional terms: a reason’s strength is measured by how often it results in the relevant form of epistemic activity in agents that it favours. An agent ‘overcomes’ or ‘defends against’ reasons when they fail to engage in the epistemic activity in the manner that the provided reasons support.^13^ On this understanding, propaganda based on lies and containing numerous fallacies can still count as a strong argument, while a mathematical proof with high support from expert mathematicians can count as weak for a particular agent.

The immunisation that results from inoculation need not be absolute or permanent. Again, the medical context is instructive. For example, influenza viruses can mutate in ways that enable them to get past the body’s defences, even if one has previously been vaccinated. Some vaccinations can have reduced efficacy after a certain period, which is why we have ‘booster’ shots. Additionally, vaccination can come in degrees. Some people who are vaccinated against the COVID-19 virus end up contracting it, but they experience symptoms that are much less severe than they otherwise would be. The same principles apply to epistemic inoculation too. A new argument might be successful at making someone change their beliefs where others had failed. Inoculation might not completely prevent someone from believing something, but it can significantly reduce their likelihood of believing it, their credence, or reduce the number of circumstances in which they would come to believe it. People might need to be repeatedly inoculated against bad beliefs over time to maintain epistemic immunity to those beliefs.

Scientists have conducted studies demonstrating that exposure to weak arguments does cause people to be more likely to later refute stronger arguments favouring the same conclusion, referring to this phenomenon simply as ‘inoculation’. A wide body of research shows inoculation is effective against misinformation (see Banas & Rains 2010, pp. for a meta-analsis). Rather than debunking falsehoods that people already accept, the aim is to ‘prebunk’ (Fazio et al., 2015) those falsehoods, stopping them from spreading in the first place. This helps to counteract the ‘continued influence effect’, in which retractions and corrections fail to result in people ‘correcting’ their beliefs, or failing to discount the effect of the original source (Lewandowsky et al., 2012). It also helps mitigate against the phenomenon where the more exposure people have to a claim they know is false, the more likely they are to end up believing it (Fazio et al., 2015). Inoculation has been shown to be effective regarding beliefs on many matters, including topics within public health (Maibach & Parrott, 1995; Niederdeppe et al., 2015), political campaigning (Burgoon et al., 1987) and climate change (Cook et al., 2017; van der Linden et al., 2017). It can also enhance the strength of other arguments. For example, while telling people about the scientific consensus on climate change can increase acceptance that climate change is real, this can be undermined when audiences receive a countervailing message. But with inoculation, this undermining effect does not occur (van der Linden et al., 2017).^14^

While this is a useful body of research, careful attention reveals that the term ‘inoculation’ in this literature is disanalogous to medical inoculation because the immunity that results doesn’t always come from exposing people to weak arguments and having them overcome those arguments. Immunity in these studies is sometimes produced simply as a result of warning subjects about misinformation they might encounter in the future, persuading them of a certain position, or correcting false beliefs that subjects already possess prior to the experiment. Begby (2021) notes that people can be influenced to maintain their beliefs in the face of contrary evidence as a result of what he calls ‘evidential pre-emption’, where a speaker warns a hearer about some later, apparently contrary evidence, in order to suggest that the later evidence is misleading or has already been taken into account.^15^

Let’s pause to consider how to best use our terms. One option is to reserve the term ‘epistemic inoculation’ specifically for cases featuring weak reasons or arguments which the agent overcomes themselves. We could instead refer to other immunity-boosting processes such as persuasion and education as ‘epistemic immunisation’, and say that some of these scientists researching ‘inoculation’ are either conceptually confused, failing to notice the limits of their analogy, or using misleading descriptors.

Against this proposal, it does seem apt to our ears to describe someone who has been brainwashed as having been inoculated against certain beliefs, despite this not necessarily resulting from exposure to weak arguments and overcoming them. Additionally, using the term ‘immunisation’ to refer to immunity-producing processes that don’t involve overcoming weak reasons risks being too broad. For example, we end up having to say that by trying to run five miles and struggling, we have been immunised against believing marathons are easy, which seems like a bit of a stretch.

The term ‘immunisation’ places less emphasis on a particular property we would like to draw our audience’s attention to, which we think these scientists are tracking, and which ‘inoculation’ is more effective at invoking. We’d like to propose that ‘inoculation’ has some associations in English that are worth highlighting. The first is that immunity is produced. The second is that the immunity is a product of exposure to something that was introduced. The in- prefix, in the context of *in*oculation, means “in, into, towards or within” (such as insert, induce, intrude; Macmillan Dictionary, n.d.). The third is that the introduced thing was introduced *by* others, i.e. inoculation is something that we *do* to people. While ‘immunisation’ has the first of these associations, it only sometimes has the second or the third, often being provided by context or other linguistic markers. This is because—though medical inoculation is a form of immunisation—immunisation also refers to someone developing immunity as a result of their immune system learning to defeat a pathogen, or using antibodies from someone else, such as when a foetus gains immunity from its mother’s antibodies. In both of these cases, immunity is not directly produced as a result of others introducing something, rather, it just ‘happens’ to the agent.

It’s this third association we want to emphasise. The proposal here is that, when providing an explanation for how someone’s epistemic immunity resulted, the factors we point to as relevant can be more or less social in nature. Factors that are more social, contingent, or which we collectively have more control over, are more fitting of the term ‘inoculation’.^16^ Becoming immune to thinking marathons are easy is the kind of thing which almost any ordinary argent who struggles to run will develop; there’s no need to point to social and cultural factors as part of our explanatory story for how that agent became immune. It’s not something we *do* to the agent. In contrast, becoming resistant to this belief as a result of indoctrination and constant propaganda does seem more inoculation-like, and that is because there is a much stronger social component in our explanation.

Whether our chosen explanation emphasises social factors is likely to be highly context-sensitive, differing according to our interests, kind of inquiry, and some assumptions about what is normal. For example, you might think that someone’s resistance to believing marathons are easy is, in fact, socially produced, because they were unjustly prohibited from receiving marathon training that everyone else was given. But this is simply part of the nature of causal explanations: which one is most relevant often depends on context (Van Fraassen, 1980). As this paper is considering the epistemic health of various parties, how this can best be promoted and researched, and what things are currently contributing to good or ill health, we have a principled reason for weighting this association strongly when assessing which terms are going to best communicate our ideas. We take it that this usage will not be confusing, given the aforementioned scientists researching ‘inoculation’ already adopted it accidentally.

We thus propose that epistemic inoculation be understood more broadly than an analogy with medical inoculation might suggest. Epistemic inoculation occurs when one’s epistemic immunity to engaging in certain epistemic activities is produced as a result of social (including cultural and political) processes. Since so much of what we learn is passed down through institutions and culture, there’s a lot of inoculation about for us to study. With this broader conception on the table, let’s now flesh out the idea in more detail. It will help to contrast our understanding with how scientists researching ‘inoculation’ typically characterise the phenomenon they are interested in, as their understanding is narrower than ours in a number of ways.

The first way existing scientific research is too narrow concerns *what* we are inoculating people against. Most studies focus on inoculating against forming particular kinds of *beliefs*, which are taken to be unhealthy in virtue of their content being false.^17^ But, given epistemic health is about much more than just beliefs, we can inoculate against other things too. For example, we can inoculate against certain attitudes and objects, such as *trusting* particular *sources*. Given someone has made arguments or reported stories which we can identify as poorly-supported or poorly-sourced, you’re going to be much more likely to dismiss future arguments or stories from them which are in fact much more well-supported.^18^ Agents can be inoculated against making certain kinds of *inferences*: teaching fallacies in a critical thinking unit aims at exactly this.^19^ Communities can be inoculated against certain kinds of *ideologies*.^20^ After being exposed to a number of bad arguments in support of, say, communist land rights and communist governance, a community might be more inclined to also reject arguments in favour of communist economies, even if they haven’t previously been exposed to arguments for communist economies specifically.

It is also possible to inoculate against ideas, positions, sources etc. that have certain kinds of qualities. For example, a proposal might be too utopian, too absolutist, or even just too complicated, which might be relevant when public understandability is important. We can also inoculate against certain kinds of attitudes or dispositions, such as arrogance, dismissiveness, or being uncharitable. This can happen in two directions. One might, as a result of being exposed to uncharitable speakers and finding them to be unconstructive interlocutors, become more likely to reject claims made by future uncharitable speakers. Alternatively, one might become less likely to be uncharitable themselves as a result of having been on the receiving end of such treatment and finding it distasteful.

A third way existing scientific research is too narrow is that it mostly takes individuals to be the relevant unit of analysis and focal point for providing explanations. But of course, much of our present interest in this phenomenon stems from observing how large proportions of communities can become epistemically unhealthy. Understanding the roles that certain factors play is best served by taking a broader scope of inquiry. Just as we can better understand an individual’s physical health-relevant choices by expanding our scope to consider social, environmental, political and cultural factors, as public health researchers do, researching epistemic health will, in some cases, require us to consider units of analysis that are larger than particular individuals, and factors beyond the moment at which they accept or reject an argument. We can even think about the inoculation of group agents, such as institutions or corporations, which typically have a structured decision-making procedure (List & Pettit, 2011). For example, exposing a corporation to bad arguments for a proposition which are then rejected might make the board members much less likely to later vote for policies supporting a similar proposition, even after stronger arguments have been presented, and even if individual board members prefer those stronger propositions.^21^

The final difference we want to note between existing scientific research and our proposal concerns the *way* in which inoculation against a position, belief, ideology etc. produces immunity. Suppose a community is exposed to arguments to solve poverty by printing money, finds them convincing and begins printing money, quickly realises inflation is terrible, and goes back to their old policy. Suppose the community as a whole recognises that calls to print money regularly *sound* persuasive, and is worried they may not be able to resist calls to print money in the future, because the current problems will be a distant memory and those future calls might be accompanied by more sophisticated arguments. The community might create immunity against this by banning arguments in favour of printing money made from being made in public spaces. Recall that people are sensitive to factors such as the number of people they see supporting a position, which can act as higher-order evidence of the truth of some propositions. Part of the way this example’s society is maintaining its immunity is by removing the influence of such factors. Even if we as citizens encounter other citizens in private spaces making arguments for that proposal, without seeing that view expressed in public spaces we might think that it is only a fringe view when in fact it is widely shared. Policies which restrict public expressions of support for that position might prevent us from e.g. having certain forms of common knowledge, which can radically affect the payoffs or rationality of various options (Aumann, 1976; Geanakoplos, 1992). Given the defences of this community against the proposal to print money were developed as a direct result of exposure to bad arguments to print money, and overcoming those arguments, this seems to be an instance of epistemic inoculation. This is despite there being a very different mechanism to the kind of immunity-sustaining mechanisms in individual agents.

## The Risks of Epistemic Inoculation

Now armed with these concepts, it can be tempting to want to go out and begin improving other people’s epistemic health, in particular, by trying to inoculate them against various epistemic activities thought to be unhealthy. While we agree this is a valuable goal, in this final section we want to identify two important risks that are worth considering before engaging in such a project.

The first risk is that is that agents can also be made immune to *healthy* beliefs, inferences, authorities, and ideologies. Epistemic inoculation and epistemic immunity-boosting processes are not just a phenomenon, they are also *tools* which can be used in immoral or epistemically unhealthy ways. Being aware of their effect is important because once someone is immune to certain reasons, it can greatly limit what avenues we have to improve their epistemic health. For example, consider how difficult it would be to convince someone that climate change is dangerous while they hold fixed their belief ‘scientists cannot be trusted’ and reject most thing that conflict with this belief.

There are two main ways in which this risk can eventuate. The first is that inoculation can be used by ill-intentioned agents who aim to make audiences immune to healthy epistemic activities, because those activities are inconvenient for those agents. Consider the large number of people who believe that climate change is not occurring, or is not caused by humans or is not worth worrying about (Flynn et al., 2021; Leiserowitz et al., 2021). A number of these people have plausibly become immune to trusting climate scientists through various forms of inoculation. The most notable method is through exposure to arguments from ‘scientists’ who are, in fact, not scientists (or not scientists with relevant expertise), but pretending to be. Such faux experts are often hired by agents or organisations who intend to distort the facts (Oreskes & Conway, 2010) or create images of false balance, which numerous studies have shown decreases audiences’ ability to accurately assess levels of consensus (Fahy, 2017; Koehler, 2016; Kortenkamp & Basten, 2015).

We’ve already commented on how a variety of factors are necessary to maintain epistemic health, and it is likely there are ways to make people immune to these more insidious attempts at inoculation (e.g. by warning people about false balance). But preventing ill-intentioned agents from being able to inoculate audiences against healthy beliefs might require more targeted measures, though assessing which ones are best all-things-considered is outside the scope of this paper. It might mean directly outlawing expressions of support for certain kinds of ideas or ideologies (e.g. Nazism), or conversely, it might mean creating strong protections for free speech to prevent censorship by ill-intentioned government officials. Different immunity-promoting policies are likely to have different trade-offs between different epistemic (and moral) goods.

Being aware of the negative effects ill-intentioned agents can have on our collective epistemic health may push us towards doing as much as we can to improve people’s health on various matters. But such impulses need to be balanced by an awareness that agents can also be inoculated against healthy beliefs by well-intentioned agents, including ourselves. For example, inoculation can occur when agents (including genuine experts) make hyperbolic or exaggerated claims which are later proven to be exaggerated. Perhaps this exaggeration stems from good motives—e.g. the agent knows that their audience is unlikely to accurately assess various risks if the agent presents their claims as accurately as possible—but it might, over the long term, lead to ignoring other accurate claims entirely. Another way it can occur is by speakers continually not taking into account the interests and values of the audience, or communicating in a condescending manner. If speakers are not signalling that they respect their audience, or are sensitive to their audience’s concerns, it can be easy for audiences to dismiss speakers as untrustworthy, and also implicate others who are similar to that speaker (e.g. other experts) as untrustworthy too.

Such risks give us reason to be careful with many of our pronouncements, to not assign fault to particular agents or groups for certain actions without double-checking we’ve got all the facts, and to temper calls to be vocal on complex social issues. They give us good reason to avoid engaging in epistemic trespassing, which occurs when one speaks confidently on matters beyond their expertise (Ballantyne, 2019). Such habits can, over the long term, end up reducing the extent to which later, more important claims receive uptake.^22^ Additionally, even if not explicitly arguing for a particular conclusion on an issue, one might increase unhealthy inoculation within one’s own epistemic community by seeking out, sharing, or talking about ideas that only represent the most extreme or fringe versions of people they disagree with.

The second risk we wish to draw attention is more subtle, but no less important for that fact. It also acts as an example of inoculation whose effects might be hard to notice without first understanding the concept of epistemic inoculation. It concerns the kinds of things which we think people are most in need of immunizing against. In critical thinking classes, philosophical research, media op-eds, and everyday conversations, when looking for examples of agents who violate various epistemic standards or who have poor epistemic health, it is common to point to conspiracy theorists, Nazis, homeopathy supporters, creationists, and climate science deniers, among others. Such examples are also reflected in the existing empirical research on inoculation, which have begun to focus on climate change deniers and (pre-COVID-19) anti-vaxxers. Let’s call this collection of agents ‘The Usual Suspects’. Sometimes, there are good reasons to use The Usual Suspects as token examples. We’ve used such examples earlier in this article because they are likely to make conceptual points understandable for our audience, other examples might feature conflicting evidence or rely on substantive issues or involve complex debates from other areas of epistemology, and things like ‘support for vaccines’ might be easier for scientists to quantify and measure than other epistemic activities. But by relying on such examples, there is a risk we are accidentally inoculating ourselves against something which contributes to good epistemic health, even in the process of arguing for why inoculation is needed.

A necessary step in being able to intentionally improve epistemic health is identifying what good epistemic health looks like and what factors contribute or degrade it. But many people’s concepts aren’t formed as a result of careful philosophical reflection, instead, they are the result of being trained on a set of examples from which people learn to notice certain features and then generalise. The worry is that we are marking out The Usual Suspects as the clearest examples of poor epistemic health when, in fact, they are not representative of many other kinds of problems that lead to poor epistemic health, or they do not *look like* the kinds of unhealthy epistemic activities that many people are most at risk of engaging in.

An example might make this clearer. It has notably been found that, compared to people who do believe in climate change or evolution, agents who are sceptical about climate change or evolution do not score any worse on questions about *what scientists believe* on these matters (Kahan, 2015). That is, what causes many people to believe or not believe these positions is not as attributable to their understanding of the subject matter as it is to their cultural identity. This seems to make many people’s belief in climate change rather precarious, epistemically speaking.^23^ But the surprising nature of this finding means that people tend not to know that their political opponents have just as much understanding as they do (and perhaps even qualify for epistemic peerhood, which traditionally is thought to give one reason to reduce their confidence when disagreement is noticed); rather, they tend to believe that the other side is uninformed, ignorant, and biased. As a result, agents can easily believe that global warming sceptics are silly while also regularly arguing that we should believe climate change is real because this weekend was very hot, and a public figure thinks it’s happening, and if we don’t believe it bad things will happen (which confuses climate with weather, appeals to an illegitimate authority, and begs the question respectively).

We believe that too much collective focus on The Usual Suspects can produce an inoculating effect. Because these are examples of people who hold beliefs that most of us and our audiences were never at much risk of believing in the first place, it leads said audiences to think that the kinds of mental habits and inferences which result in poor epistemic health are *obvious*, or stem from some abnormal personal defect that we ourselves are rarely susceptible to. Treating The Usual Suspects as the most central examples, or as the kinds of things that we most need to train ourselves to recognise, risks reducing the extent to which we examine our own epistemic health, learn to identify moments where we may be being led astray, and teach others to do the same.

By analogy, failing to brush one’s teeth will non-trivially contribute to poor physical health, given long enough. It is certainly worth educating people about. But it would be unwise, from a public health standpoint, to try to improve everyone’s overall physical health by continually pointing to dental caries as our central example of what is to be avoided. It would be unwise to build most of our theories about how to improve public health by trying to extrapolate from what we know about teeth cleaning. And it would certainly be bad if citizens got the impression that the way they prevent dental caries was how they should also prevent tuberculosis.

One of the risks of this inoculating effect is simply that we fail to believe what we have good reason to believe, or misidentify what bad epistemic health looks like. But a deeper worry concerns the attitude or default affective tone with which people end up approaching various debates. Giving too many examples of poor inferences that aren’t *tempting* for us to make can lead us to think that we’re immune to such temptation, or that maintaining good epistemic health is easy. Alternatively, it creates an impression that epistemically healthy views are almost always extremely well-supported by lots of evidence whose strength is self-evident, while false or unhealthy views are not.^24^

In short, the risk we are warning against is accidentally inoculating ourselves against good epistemic habits by ‘Othering’ poor epistemic habits and poor epistemic health; roughly, conceptualising poor epistemic habits as properties that belong to *other* social groups in a way that sets *them* apart from *us* (Rohleder, 2014).^25^ Such practices imply that the kinds the kinds of mental habits used by one’s own ‘team’ are epistemically acceptable even if they stem from unhealthy dispositions to e.g. ignore lots of research and rely on straw man arguments.

This point may appear to be in tension with the previous risk we identified: isn’t doubt part of what is causing high levels of climate scepticism? And here we instead seem to be saying that we should be less confident in our ability to understand climate science. But the very tendency to think there is a tension here is indicative of the risk we are trying to identify. We are not saying that the views of The Usual Suspects should be taken seriously, or that everyone should ‘do their own research’. We take it to be epistemically acceptable to simply ignore or dismiss arguments given by many of The Usual Suspects, and to guard against increasing one’s level of doubt in response to any points they make that seem somewhat plausible. The worry is that if The Usual Suspects are treated as *prototypes* of bad reasoning or poor epistemic health, we teach people that the epistemically appropriate response in other debates we find silly or untempting is to ignore those interlocutors and guard against doubt there too. But it’s precisely because many other debates are different in terms of the nature of the evidence available and the epistemic character of one’s interlocutor that they call for different responses.

The appropriate response to this risk we are identifying is not to simply strike a balance between doubting too much and not doubting enough (though this is prudent advice). It is to ensure we are not accidentally inoculating ourselves against *healthy doubt* and other healthy attitudes. For example, we need to avoid inoculating ourselves and others against understanding that scientific evidence is often complicated, with conflicting data, and many things currently in peer-reviewed journal articles will later turn out to be wrong. Or against the idea that sometimes ideas we find intuitively absurd or repugnant at first will later turn out to be true, or that we may be living in epistemic bubbles in which many people we trust are incorrect about certain matters (Nguyen, 2020). We also need to not inoculate against recognising those instances in which we *should* do (some) research for ourselves, if only to check whether the relevant body of experts has a consensus on some matter, or whether there are useful base rates with which to contextualise certain statistics we come across.

How exactly to distinguish between instances in which being dismissive of someone we disagree with is epistemically acceptable, and instances in which it is not, is an important question outside the scope of this paper. Identifying what kinds of mental habits we are most at risk of adopting, or already in need of improving, is also an important question we will must leave for another time.^26^ But hopefully our audience is not immune to noticing that the more frequently people assume large swathes of society are immune to reason, the more likely it is they are indirectly contributing to this becoming the case.

## Conclusion

In 2016, politician Michael Gove was interviewed on whether leaving the EU would be good for the UK, given a very large number of institutions and experts had said it would be bad in a number of ways. In support of leaving, Gove famously quipped on television that “the people of this country have had enough of experts” (Mance, 2016). Though he later clarified he was restricting his comments to particular institutions like the IMF, and their forecasts about how trade benefits would be distributed (BBC News, 2017), the comment sparked a lot of debate about the current relationship between citizens and epistemic authorities, and many voters seemed to endorse Gove’s overall sentiment.

We won’t comment on the Brexit vote specifically, or the beliefs of those hesitant to get vaccinated against COVID-19. But a state of affairs in which large numbers of people no longer trust experts is the kind of thing social epistemologists ought to be interested in beyond assessing its rationality, reasonableness, justness, or epistemic virtuousness. Such states of affairs are the kind of thing likely to undermine a variety of epistemic goals we have. Epistemic health allows us to see many different parts of society and types of inquiry as contributing to shared goals, and gives us a language for assessing how well different kinds of entities are functioning with regard to those goals. Lack of trust—and many other activities of interest—can manifest as a form of resistance unlikely to be solved by simply giving people more information. Epistemic immunity allows us to think about forms of resistance to certain kinds of epistemic activity, which can contribute to or undermine one’s epistemic health. Finally, such resistance doesn’t come out of nowhere. It was produced by particular social, cultural and political factors that are worth investigating and understanding, so that they can then be countered. Epistemic inoculation draws our attention to the ways in which we produce such immunity, good or bad, in others and ourselves.

While these are useful concepts, attempts to improve others’ epistemic health come with risks, even if well-intentioned. It can be easy to think that the best way to improve epistemic health is to protect people against the most obvious instances of unhealthy epistemic activities. But such an attitude may inadvertently lead us to immunise one another against the wrong thing, particularly if the things taken to be obvious are not those that are most common, important, or that we are most at risk of committing. Such a predicament is not therefore a call for increased doubt. Rather, it is a call to recognise that improving one’s epistemic health is very unlikely to feel like avoiding epistemic activities one already finds unintuitive. The more time we spend trying to promote epistemic health by focusing on and extrapolating from positions we were never at much risk of believing, the more time we waste not improving our current unnoticed and unhealthy habits.

## Footnotes

1 Save for Merritt on what Kant means, but his broader project is quite different to ours.

2 Daniels (2007, p. 42) understands health as normal species functioning, though he doesn’t describe it in terms of necessary and sufficient conditions.

3 Such points are commonly used to argue for normativism/constructivism, the position that health is an inherently normative concept (Murphy, 2021).

4 Philosophers who argue health can be a purely descriptive concept—and whose interest in health stems from examples like these—are known as naturalists (e.g. Boorse 1977). Though the debate between normativists and naturalists is very complex and contentious, see Griffiths and Matthewson (2018) for a more recent defence of how health, disease and dysfunction can be understood in non-normative terms.

5 Given the ease with which we can understand what we are talking about here before having spelled out the details, it is an open question whether we should think of this as a brand new concept which shares some features with health, or a concept we already possess to some degree which is simply being pointed out to us. Even if we are only providing a new term for an existing concept, our main interest is in the concept’s potential roles which have not previously been analysed.

6 A number of philosophers have tried to argue that some beliefs have more value than others (e.g. Goldman 1999; Alston, 2005; Kelly, 2003), for instance, though others (e.g. Lynch, 2004) argue that all beliefs have *prima facie* value.

7 Alfano (2021), for instance, has looked at how topologies of networks can be more or less conducive to successful inquiry, independent of the dispositions of agents in those networks. These topologies can be invoked in explanations of why different networks produce different epistemic behaviours in agents. The spread of misinformation, for instance, has been cited as a contributory cause to the election of various leaders in some countries, and it seems plausible that otherwise similar countries might have avoided similar results by having more healthy epistemic networks.

8 Additionally, Aristotle takes functioning to concern the manifestation of virtuous *activity*, not solely the presence of the disposition, whereas we are quite happy to attribute both physical and epistemic health to someone even when e.g. they are sleeping with little activity occurring. Thanks to [Redacted] for helpful discussion.

9 What counts as unhealthy, threatening or pathogenic is subject to debate, and we won’t take up the issue here. An anonymous reviewer asks whether our characterisation of epistemic immunity is simply Battaly’s (2018) account of close-mindedness under another name, given she characterises close-mindedness as an unwillingness or inability to engage seriously with intellectual options. We think there are a number of significant differences between the two accounts, which further points towards substantive differences between our framework and virtue epistemology: (i) Agents can be close-minded without being very immune. Someone might take themselves to be dismissive of *P*, and treat people who believe *P* as silly, thus being close-minded. But because the way that our beliefs update is often not transparent to us, and can change even when we do not engage seriously with an idea, their beliefs might unconsciously change over time, as e.g. they get cues that people of their in group believe *P* (Levy, 2021). They would thus be not very immune to believing *P*. (ii) Agents can be immune without being close-minded. Battaly argues that most reasonable agents who refuse to take seriously the proposition that the holocaust never happened are not being close-minded, as there are some relevancy restrictions on application of the concept. In contrast, we argue that such agents are immune to such beliefs (and probably glad to be so).

10 Though it might still be the case that any given application of the concept is value-laden in the sense that what the speaker considers to *count* as neutral, or to be an open question, can reflect certain values and assumptions.

11 Vaccination is a kind of inoculation. People used to inoculate against smallpox by transferring matter from scabs or pustules, but these weren’t vaccines. The term ‘vaccination’ used to apply only to cowpox inoculation (‘vacca’ being Latin for ‘cow’) but today the term applies much more broadly to prepared products that provide active acquired immunity.

12 A common misconception is that the vaccine was made from cowpox virus, as this was what Jenner first proved can inoculate against smallpox. Subsequent research suggests the vaccinia virus is more closely related to the horsepox virus, though all four viruses have a common ancestor. See Esparza, Schrick, Damaso, and Nitsche (2017).

13 This characterisation may need to be refined slightly, to exclude cases where e.g. the hearer simply didn’t notice or understand the reasons being provided. Note also an agent might have taken themselves to have overcome an argument, but later come to accept it without realising. It is possible that differing theoretical considerations favour narrower or broader understandings of what counts as epistemic inoculation. We aim to be ecumenical in this paper, and below we offer some examples of what might count as epistemic inoculation.

14 Scientists have been aware of the general phenomenon for several decades, but earlier experiments focused on statements taken from common platitudes rather than substantive disagreements (Papageorgis & McGuire, 1961).

15 Thanks to an anonymous reviewer for this source.

16 Absences or omissions can count as part of a causal explanation, and can be socially produced.

17 While some research examines subjects’ attitudes, these have been limited to degree of support. An exception is Jackson, Compton, Whiddett, Anthony, and Dimmock (2015) who found it is possible to inoculate self-efficacy (understood as confidence in one’s ability) against negative feedback.

18 Or if you’ve previously experienced evidential pre-emption, having been warned about a source of evidence that seems to conflict with someone’s assertion.

19 Though how effective critical thinking or philosophy courses are at generating skills which transfer to tasks beyond those specific classrooms is dubious. See El Soufi and See (2019); Huber and Kuncel (2016); cf. Ortiz, 2007.

20 Some have proposed that certain ideas or memes (in Dawkins’s (1993) sense) should be understood as analogous to viruses in a more literal sense, in that they have purposes of their own, are shaped by (cultural) evolutionary processes, and their success in replicating comes at the expense of the host’s interests (e.g. Boudry & Hofhuis 2018). We’ll set this question aside.

21 Note that individual members don’t always vote according to their personal preference. For example, they might vote according to what they think is required of their role, or according to what outcomes they think will have the largest consensus, or because certain information they possess is not yet common knowledge.

22 A more familiar example of undesirable inoculation can occur within critical thinking units. Many philosophers will have had the experience of teaching students about fallacies, and later observing them misapplying that knowledge to reject arguments that, while committing a fallacy, nevertheless give good reasons to believe a particular conclusion.

23 One might reply that this is not cause for concern if climate change believers simply have a cultural identity which is based around epistemically healthy habits, such as trusting scientists. We grant the point, but think this is likely to be overly charitable for many citizens, at least without establishing that climate change believers would continue to trust scientists even when this conflicts with other beliefs important to their cultural identity. We would need to assess whether such agents would also believe scientists if scientists were to form something of a consensus that e.g. more guns in a country results in less murders, that banning minimum wage or unions improves living circumstances for the poor, or that funding for mental health services is completely ineffective at improving people’s mental health.

24 This is another reason why the concept of epistemic health is useful—it better equips us to characterise epistemological goals and progress in positive terms, rather than simply *via negativa*.

25 Consider, for comparison, Semmelweis’s discovery that hand-washing by doctors delivering babies could drastically reduce the mortality rate of mothers giving birth, mostly because said doctors had previously contaminated their hands carrying out autopsies. Semmelweis’s findings were famously ridiculed and mocked by some doctors, partly because they felt having unclean hands was incompatible with their social status as gentlemen (Carter & Carter, 2017).

26 Plausibly, habits like regularly making testable predictions, or learning to correct for cognitive biases by e.g. learning to take into account base rates, are the kind of thing which, over time, will help people better calibrate how confident they ought to be on various matters.
